# Detection of the valvular split within the second heart sound using the reassigned smoothed pseudo Wigner–Ville distribution

**DOI:** 10.1186/1475-925X-12-37

**Published:** 2013-04-30

**Authors:** Abdelghani Djebbari, Fethi Bereksi-Reguig

**Affiliations:** 1 University of Tlemcen, Faculty of technology, BP119, Tlemcen, Algeria

**Keywords:** Phonocardiography, Heart sound, Valvular split, Reassigned Smoothed Wigner–Ville distribution

## Abstract

**Background:**

In this paper, we developed a novel algorithm to detect the valvular split between the aortic and pulmonary components in the second heart sound which is a valuable medical information.

**Methods:**

The algorithm is based on the Reassigned smoothed pseudo Wigner–Ville distribution which is a modified time–frequency distribution of the Wigner–Ville distribution. A preprocessing amplitude recovery procedure is carried out on the analysed heart sound to improve the readability of the time–frequency representation. The simulated S2 heart sounds were generated by an overlapping frequency modulated chirp–based model at different valvular split durations.

**Results:**

Simulated and real heart sounds are processed to highlight the performance of the proposed approach. The algorithm is also validated on real heart sounds of the LGB–IRCM (Laboratoire de Génie biomédical–Institut de recherches cliniques de Montréal) cardiac valve database. The A2–P2 valvular split is accurately detected by processing the obtained RSPWVD representations for both simulated and real data.

## Introduction

**Figure 1:**
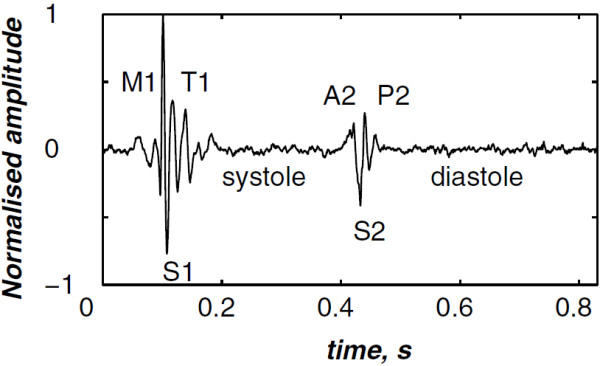
Normal PCG signal (one cardiac cycle).

Heart sounds are recorded as a digital signal to be processed by advanced digital signal processing techniques. This processing provide valuable information in relation to the cardiac activity of the patient. The acoustic recording of heart sounds also known as phonocardiogram (PCG) signal is achieved by means of a microphone placed carefully on the chest of the patient. The PCG signal is therefore composed of sounds of the heart generated during the systole and the diastole phases which are mainly marked by the first (S1) and the second (S2) heart sounds. Two others sounds denoted S3 and S4 could appear during the diastole. Thus, the PCG signal is mainly formed by the S1 and the S2 heart sounds as depicted in Figure 1. The S1 heart sound is mainly composed by 2 valvular sounds denoted M1 and T1 generated by the closure of the mitral and the tricuspid valves respectively. Likewise, the S2 heart sounds is formed by A2 and P2 components which are generated by the closure of the aortic and the pulmonary valves respectively.

It is well known that digital phonocardiography is a powerful tool for assessing the pulmonary artery pressure than Doppler echocardiography [1]. Xu *et al.*[2] found out that the pulmonary artery pressure is correlated with the A2–P2 valvular split.

The split within the S1 and the S2 heart sounds emerged as an indicator of several valvular diseases alongside the Doppler echocardiography (DE). However, the DE is inaccurate in approximately 50% of patients of normal pulmonary artery pressure (PAP), 10–20% of patients with increased PAS, and 34–76% of patients with chronic obstructive pulmonary disease, a weak Doppler signal or a poor signal to noise ratio (SNR) [2]. Indeed, Fisher *et al.*[3] studied the accuracy of the DE in hemodynamic assessment of the pulmonary hypertension (PH). They demonstrated that DE can usually overestimate and underestimate the PAP in PH patients. This can be partly explained by inaccuracies of the right atrial pressure estimation as well as poor Doppler imaging of the transtricuspid regurgitant blood flow. Moreover, Rich *et al.*[4] compared the Doppler echocardiography (DE) with the right sided heart catheterisation (RHC) as an invasive measure of the PAP in 160 patients with PH. They found out that the DE is inaccurate in estimating the PAP in 50.6% of patients at a bias of 8.0 mmHg. Therefore, the DE–based estimation of the PAP is not reliable to diagnose the PH or to assess the efficacy of therapy.

In contrast, the split duration as well as the dominant frequency of P2 are increased in pulmonary hypertension and are considered as reliable parameters to estimate the PAP. Xu *et al.*[2] found that the duration between the onsets of the aortic (A2) and the pulmonary (P2) components within the S2 heart sound (S2) allow accurate measurement of the PAP through advanced digital signal processing techniques. However, this split duration is limited (<100 ms) and still difficult to measure since these components are often overlapping and are of frequency modulated chirp behaviour [5,6]. The separation of the valvular components of both the S1 and the S2 heart sounds remains a problematic issue. Indeed, several studies reported the complexity of analysing such a transient signals formed by overlapping chirps [7,8]. Xu *et al.* proposed a nonlinear transient chirp model to simulate the A2 and P2 components of the S2 heart sound [5]. They also proposed a dechirping approach using the Wigner–Ville distribution (WVD) to estimate the instantaneous frequency (IF) of the aortic (A2) and the pulmonary (P2) components. However, they reported weak energies at the beginning and the end of each chirp component to recover the frequency modulated behaviour of heart sounds in the time–frequency plane. This is due to the weak amplitude of all of the valvular heart sound chirps at their onsets and their ends [5].

The A2–P2 valvular split can be originated under physiological or pathological conditions. In normal subjects, a physiological split of the S2 heart sound can occur during inspiration as a result of the delayed pulmonary pressure to raise over the right intraventricular pressure which closes the pulmonary valve. Cardiac pathologies such as the right bundle branch block and the pulmonary stenosis may induce a wide S2 split [9,10].

In a previous paper [11], we applied the Smoothed pseudo Wigner–Ville distribution (SPWVD) on aortic stenosis and normal heart sounds to quantify their different spectral content within the time–frequency plane. The time-frequency representations we previously obtained by the SPWVD yield global quantification of the valvular intracardiac activity and should be improved through reassignment [12,13] to adequately represent the intracardiac valvular activity within each heart sound.

Santos *et al.*[14] proposed an A2–P2 valvular split detection algorithm based on the instantaneous frequency calculated by the Hilbert transform. However, the instantaneous frequency of a multicomponent signal cannot be estimated as the derivative of the phase of its analytic signal which is calculated through the Hilbert transform [15]. Therefore, a powerful method that takes into account the multicomponent behaviour of heart sounds should be investigated to represent the intracardiac valvular activity.

In this paper, We developed a new algorithm based on the Reassigned smoothed pseudo Wigner–Ville distribution (RSPWVD) to accurately detect the A2–P2 valvular split within simulated and real S2 heart sounds. Firstly, we recovered the onset and the end amplitude of each valvular component by an envelope recovery procedure we developed. Secondly, we reconstructed the IF of simulated heart sounds at a higher time–frequency resolution by the RSPWVD. We recovered their time–frequency content at several valvular split durations (from 30 to 60 ms at a step of 10 ms). Subsequently, we processed real S2 heart sounds of the LGB–IRCM (Laboratoire de Génie biomédical–Institut de recherches cliniques de Montréal) cardiac valve database to validate the algorithm in real conditions.

This paper is organised as follows. Section “Phonocardiographic data”, entitled ‘Phonocardiographic data’, presents simulated and real S2 heart sounds to be processed by the developed algorithm. Section “Time–frequency analysis”, recalls the theoretical background of the WVD, Smoothed pseudo WVD (SPWVD) and the Reassigned SPWVD (RSPWVD). Section “Detection algorithm of the A2–P2 valvular split” presents the detection algorithm of the A2–P2 valvular split within S2 heart sounds. Sections “Detection of the A2–P2 valvular split in simulated heart sounds” and “Detection of the A2–P2 valvular split in real S2 heart sounds of the LGB–IRCM cardiac valve database” present the detection results of the algorithm applied on simulated S2 heart sounds at various valvular split durations and real S2 heart sounds (LGB–IRCM cardiac valve database) respectively.

## Phonocardiographic data

### Valvular heart sound model

Tran *et al.* developed a heart sound simulator by combining a set of equations to model several phonocardiogram behaviours [16]. This model is formed by a linear chirp with an amplitude adjusted according to clinically recorded S1 and S2 heart sounds. Xu *et al.* extended this model to a narrow–band non–linear chirp signal with a fast decreasing IF over the time–frequency plane to model the valvular heart sound [5,6]. This decreasing frequency behaviour is generated by the decaying aortic and pulmonary pressures after the end of systole and during the beginning of early diastole. The modulated frequency content of the valvular sound is of chirp nature rather than linear. Indeed, in previous works, we confirmed that heart sounds are narrow–band non–linear chirp signals [17,18].

Xu *et al.* discussed the exponentially damped sinusoid model [19,20], the matching pursuit method [21,22], and the linear chirp model as modelling approaches of heart sounds. They found out that the transient nonlinear chirp signal they developed is the suitable model for the analysis–synthesis of the valvular heart sounds.

We used the model proposed by Xu *et al.*[5,6] to generate simulated valvular sounds to study the performance of the detection algorithm we developed. The valvular non–linear chirp model is defined by an amplitude and a phase functions according to (1) as follows:

(1) $$v(t)=a(t)\text{sin}\left(\varphi (t)\right)$$

where *a*(*t*) and *φ*(*t*) represent the IF and the phase of the valvular sound respectively.

**Figure 2:**
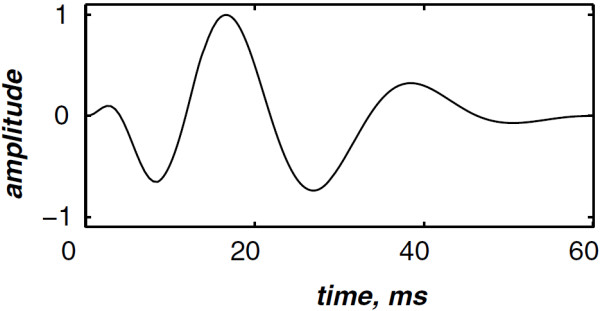
Simulated aortic (A2) valvular chirp.

**Figure 3:**
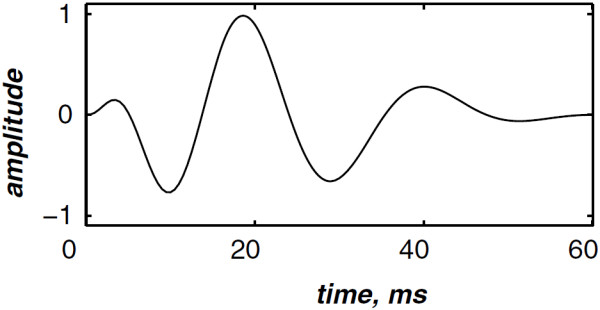
Simulated pulmonic (P2) valvular chirp.

The highest and the lowest frequencies of the chirp model differ from each valvular sound to another. However, the valvular model is valid for the overall valvular components of both the S1 and the S2 heart sounds. According to (1), Figures 2 &3 illustrates the A2 and P2 valvular sounds respectively. The A2 chirp begin from 250 Hz and falls to 53 Hz at 60 ms whilst the P2 chirp goes from 200 to 50 Hz [5,6,23]. The A2 and P2 components last usually 30 up to 60 ms but less than 80 ms [5,6]. The split duration between them rises during inspiration to reach 30 up to 80 ms, and decreases under 15 ms during expiration [24,25].

According to the valvular model in (1), the S2 heart sound is given by;

(2) $$S_{2}(t)=a_{A2}(t)\text{sin}\left(\varphi_{A2}(t)\right)+a_{P2}(t-d_{s})\text{sin}\left(\varphi_{P2}(t-d_{s})\right)$$

where (*a*_*A*2_(*t*),*φ*_*A*2_(*t*)) and (*a*_*P*2_(*t*),*φ*_*P*2_(*t*)) denote the amplitude and the phase of the A2 and the P2 valvular sounds respectively. The split duration interval denoted by *d*_*s*_ separates the beginning of A2 and P2 components. The simulated valvular non–linear chirp component duration is set to 60 ms [5].

### The LGB–IRCM cardiac valve database

The PCG signals of the LGB–IRCM cardiac valve database were recorded in the IRCM (Institut de recherches cliniques de Montréal) and the Montreal General Jewish Hospital in Quebec (Canada). This database is set up with PCG and ECG signals recorded from 172 patients with a prosthetic heart valve in the aortic or the mitral position. All patients signed an inform consent form attesting their assent to take part in the recording of the LGB–IRCM cardiac valve database.

The patient was placed in dorsal decubitus in a recording room. The back of the bed was raised to have 45° between the bust of the patient and the horizontal plane. After that, a thorax auscultation had been carried out to localise the auscultation areas on the chest of the patient. Subsequently, the recording was carried out after 5 minutes rest and calm breathing.

A precordial multi–sites recording was carried out from the aortic, pulmonary, left ventricular, and apical auscultation areas. For each PCG recording, the ECG (derivation II) signal was simultaneously acquired.

**Figure 4:**
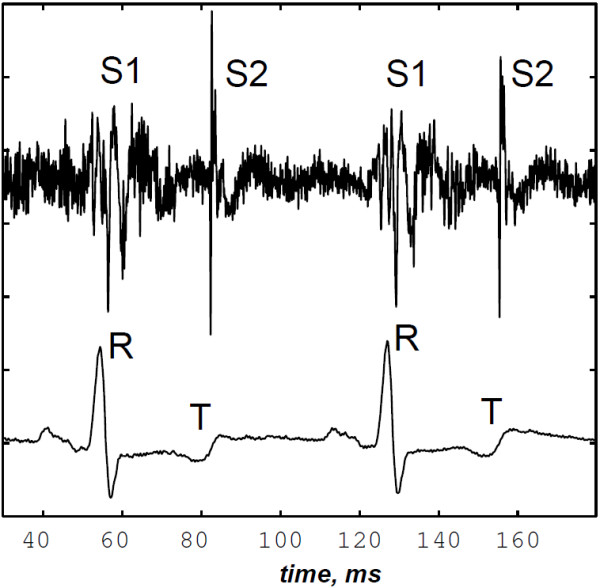
Data file sample (10001.11) of the LGB–IRCM cardiac valve database: PCG (recorded from the aortic auscultation area) and ECG signals over 2 cardiac cycles.

The PCG signal was band–pass filtered between cut–off frequencies of 50 and 2 kHz. The PCG and ECG signals were digitised by a 12 bits analog–to–digital converter on IBM–PC computer at sampling rates of 5 kHz and 500 Hz respectively. The ECG was recorded to be used as a reference signal in segmenting the PCG signal in systole and diastole phases. Each recording contains approximately 30 cardiac cycles. Figure 4 illustrates a PCG–ECG sample of the LGB–IRCM cardiac valve database.

## Methods

### Time–frequency analysis

The Wigner–Ville distribution (WVD) and the Smoothed pseudo WVD (SPWVD) are both members of the shift–invariant class, also known as the Cohen’s class. This generalised time–frequency class can be formulated as [12,26];

(3) $$C_{x}(t,\omega )=\overset{+\infty }{\underset{-\infty }{\iint }}\phi_{\text{TF}}(u,\Omega )\;WV_{x}(t-u,\omega -\Omega )\;du\;\frac{d\Omega }{2\pi }$$

where *x*(*t*) represents the signal to be analysed and *ϕ*_*TF*_(*u*,*Ω*) denotes the kernel of the time–frequency distribution.

#### Wigner–Ville Distribution (WVD)

The Wigner–Ville distribution (WVD) [27] is defined by

(4) $$W_{x}(t,f)=\overset{+\infty }{\underset{-\infty }{\int }}x\left(t+\frac{\tau }{2}\right)x^{*}\left(t-\frac{\tau }{2}\right)e^{-j2\mathrm{\pi f\tau}}d\tau$$

where *x*(.) denotes the signal to be analysed.

In order of highlight the benefits behind using the WVD, it is worthy to compare it with the spectrogram which is the squared magnitude of the Short–time Fourier transform (STFT) of a given signal *x*(*t*);

(5) $$S_{x}(t,\nu )={\left|\overset{+\infty }{\underset{-\infty }{\int }}x(\tau )h^{*}(\tau -t)e^{-j2\pi \nu \tau }d\tau \right|}^{2}$$

where *h*^∗^(*t*) is the sliding window.

The spectrogram is calculated by a linear then a bilinear operations. Firstly, the linear operator consists of a Fourier transform, and secondly the squared modulus as a bilinear operator is applied to the signal to be analysed. In contrast, the WVD begins with a quadratic estimation of the energy and then a Fourier transform is applied to the signal according to (4) [28]. The WVD combines the time and the frequency representations with some required properties to adequately represent a given signal *x*(*t*) in the time–frequency plane [27]. A summary of these nice properties can be found in the appendix in section* “Appendix: Properties of the Wigner–Ville distribution”.

The analytic form of the analysed signal is necessary to avoid the interference between positive and negative frequency components. However, cross–terms within the WVD are unavoidable between components of the analysed signal. For a multicomponent signal *x*(*t*) formed by 2 components *x*_1_(*t*) and *x*_2_(*t*), the WVD can be written;

(6) $$W_{x}(t,f)=W_{x_{1}}(t,f)+W_{x_{2}}(t,f)+2ℜW_{x_{1}x_{2}}(t,f)$$

where $W_{x_{1}}$ and $W_{x_{2}}$ are auto–terms, also know as signal–terms, are the WVDs of *x*_1_(*t*) and *x*_2_(*t*) which are assumed to be analytic as well as *x*(*t*). Whereas the term $W_{x_{1}\cdot x_{2}}$ represents cross–terms, also known as outer–artefacts, which appear midway between $W_{x_{1}}$ and $W_{x_{2}}$ within the time–frequency plane. Therefore, the WVD of *x*(*t*) is formed by the WVDs of its constituents and the cross–terms which represents their cross–Wigner–Ville distribution (XWVD). Unfortunately, when the analysed signal contains several components, the obtained time–frequency representation becomes unreadable.

#### Smoothed Pseudo Wigner–Ville Distribution (SPWVD)

The SPWVD reduces the unwanted cross-terms of the WVD by two–dimensional low–pass filtering. This smoothing is achieved by a double convolution in time and frequency by two functions *g* and *h* through the kernel *ϕ*_*TF*_(*u*,*Ω*) = *g*(*u*)*H*(*Ω*) according to (7) [29];

(7) $$\text{SP}W_{x}(t,\nu )=\overset{+\infty }{\underset{-\infty }{\int }}h(\tau )\overset{+\infty }{\underset{-\infty }{\int }}g(s-t)x\left(s+\frac{\tau }{2}\right)x^{*}\left(s-\frac{\tau }{2}\right)ds\;e^{-j2\pi \nu \tau }d\tau$$

where *g* and *h* are two real even windows with *h*(0) = *G*(0) = 1.

The SPWVD has a separable smoothing kernel (*g*(*t*),*H*(*f*)) which provides an independent control of the time and frequency resolutions. For a zero time–resolution, i.e., *g*(*t*) = *δ*(*t*), the calculated SPWVD has no time smoothing (where *H*(*f*) is the Fourier transform of *H*(*f*)). Thus, the resulting time–frequency distribution is known as the pseudo–WVD (PWVD).

Smoothing the time–frequency distribution affects the time–frequency localisation of the signal content. Therefore, a trade–off between interference attenuation and time–frequency localisation occurs to ensure a good time–frequency representation [30-34].

#### ***Reassigned Smoothed Pseudo Wigner–Ville Distribution (RSPWVD)***

The reassignment method was first applied by Kodera *et al.*[35,36] to the spectrogram to surpass its unavoidable Gabor–Heisenberg inequality [34,37] to provide a better time–frequency representation. Auger *et al.*[12] studied the reassignment method and demonstrated its effectiveness to improve the readability of all the bilinear time–frequency representations. This method rearranges the coefficients of the time–frequency distribution around new zones to yield a high resolution TFR. Thus, this method can be used as a complement to any bilinear time–frequency distribution.

The reassignment method can be formulated by recalling the generalised time–frequency representation of the Cohen’s class (section “Time–frequency analysis”). The integration within the time–frequency distribution in (3) refers to the sum of the distributions at a point (*t*,*ω*) within the time–frequency plane. This is the sum of the terms *ϕ*_*TF*_(*u*,*Ω*)*WV*(*x*;*t* - *u*,*ω* - *Ω*) which represents the weighted coefficients of the WVD in the points at the vicinity of (*x*;*t*-*u*,*ω*-*Ω*). Therefore, the distribution is concentrated at the time–frequency support of the kernel *ϕ*_*TF*_(*u*,*Ω*). Unfortunately, the cross–terms are attenuated at the cost of spreading the auto–terms of the analysed signal. The modified time–frequency distribution attributes new values to each coefficient to its neighbouring centre of gravity according to (8) and (9) as follows;

(8) $$\begin{array}{ll} \hat{t}(x;t,\omega ) & =t-\frac{\overset{+\infty }{\underset{-\infty }{\iint }}u\cdot \phi_{\text{TF}}(u,\Omega )WV_{x}(t-u,\omega -\Omega )du\frac{d\Omega }{2\pi }}{\overset{+\infty }{\underset{-\infty }{\iint }}\phi_{\text{TF}}(u,\Omega )WV_{x}(t-u,\omega -\Omega )du\frac{d\Omega }{2\pi }}\; \end{array}$$

(9) $$\begin{array}{ll} \hat{\omega }(x;t,\omega ) & =\omega -\frac{\overset{+\infty }{\underset{-\infty }{\iint }}\Omega \cdot \phi_{\text{TF}}(u,\Omega )WV_{x}(t-u,\omega -\Omega )du\frac{d\Omega }{2\pi }}{\overset{+\infty }{\underset{-\infty }{\iint }}\phi_{\text{TF}}(u,\Omega )WV_{x}(t-u,\omega -\Omega )du\frac{d\Omega }{2\pi }}\; \end{array}$$

The reassigned time–frequency representation is then formulated by (10) as follows;

(10) $$\text{RTFR}(x;t^{\prime },\omega^{\prime })=\overset{+\infty }{\underset{-\infty }{\iint }}\text{TFR}(x;t,\omega )\delta \left(t^{\prime }-\hat{t}(x;t,\omega )\right)\cdot \delta \left(\omega^{\prime }-\hat{\omega }(x;t,\omega )\right)dt\frac{d\omega }{2\pi }$$

where *δ*(.) denotes the Dirac impulse.

The reassigned time–frequency distribution is not bilinear. However, it is time and frequency shift–invariant, and respects the energy conservation property. Moreover, its powerful property perfectly localises chirps [12].

The reassignment method [13] applied to the SPWVD modifies values of the time–frequency representation as follows: 

1. Compute the Smoothed pseudo–WVD of the signal,

2. Evaluate the local centres of gravity *t*_*a*_(*t*,*ν*) and *ν*_*a*_(*t*,*ν*) of the calculated SPWVD in every point of the time–frequency representation,

3. Assign the energetic content to the new point within the time–frequency plane according to (11);

(11) $${\left|F_{x}(t,\nu )\right|}^{2}\to S_{x}\left(t_{a}(t,\nu ),\nu_{a}(t,\nu )\right)$$

Therefore, the reassignment method improves the readability of the calculated time–frequency representation by boosting the time and frequency resolutions [12,13,38].

### Detection algorithm of the A2–P2 valvular split

Xu *et al.* demonstrated that heart sounds are formed by overlapping chirp components which are generated by the closures of the intracardiac valves [5,6]. Unfortunately, the restrictive weak amplitude at the onset and the end of each chirp component confined the time–frequency chirp shape of each component at its highest amplitude domain [5].

The algorithm we developed sorts out this downside by recovering these weak amplitude areas through an envelope recovery of the analysed signal. This is the first step of the algorithm which paves the way to make up the full content of heart sounds in the time–frequency plane. The detection algorithm of the valvular split within heart sounds is summarised as follows; 

1. Envelope recovery: the signal is consecutively multiplied with its complement to 1 until the correlation between the latest consecutive signals exceeds 99.90%.

2. Calculation of the Reassigned–SPWVD (RSPWVD) of the signal.

3. Estimation of the IF by detecting frequencies of the RSPWVD coefficients with highest intensities over the time domain.

4. Processing the IF as unidimensional curve to detect the split inflection by localising the maximum and the minimum having the maximum amplitude difference.

We propose a detection approach of the A2–P2 valvular split based on the calculation of the Hilbert transform envelope of the S2 heart sound. This step is optional used comparison purposes with the RSPWVD–based algorithm and is summarised as follows; 

1. Calculation of the envelope of the signal by the Hilbert transform which is given by to (12) [27];

(12) $$\begin{array}{lll} H\left\{x(t)\right\} & =x(t)*\frac{1}{\mathrm{\pi t}}\; & \\ & =\frac{1}{\pi }\mathrm{p.v.}\left\{\overset{+\infty }{\underset{-\infty }{\int }}\frac{x(\tau )}{t-\tau }d\tau \right\}\; \end{array}$$

where p.v.{.} denotes the Cauchy principal value.

2. Detection of the split by localising the midway local minimum of the envelope at the higher amplitude.

The discrete IF is calculated as the inverse of the detected periods of heart sounds through the time domain. This detection is carried out by localising time–instants of zero–crossings of the analysed heart sounds. This discrete IF sequence enables to back up the discussion of the final IF detected from the RSPWVD.

## Results and discussion

Firstly, we applied the algorithm we developed on simulated heart sounds at various A2–P2 valvular split durations to show its ability to retrieve these splits within the time–frequency plane. Secondly, we processed several real S2 heart sounds of the LGB–IRCM cardiac valve database presented in section “The LGB–IRCM cardiac valve database” to validate the algorithm in real conditions. Time–frequency representations and the A2–P2 split detection results for a sample data file selected from the LGB–IRCM cardiac valve database are presented. The Hilbert envelope detection approach (section “Detection algorithm of the A2–P2 valvular split”) and the discrete IF are used as complementary tools to check the obtained results.

### Detection of the A2–P2 valvular split in simulated heart sounds

**Figure 5:**
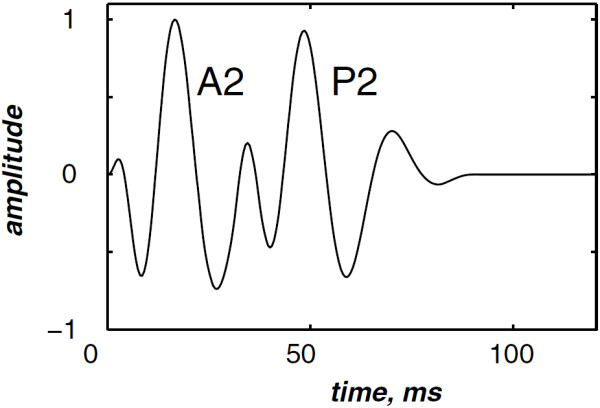
Simulated S2 heart sound (duration: 90 ms, A2–P2 valvular split: 30 ms), A2–P2 valvular split: 30 ms.

**Figure 6:**
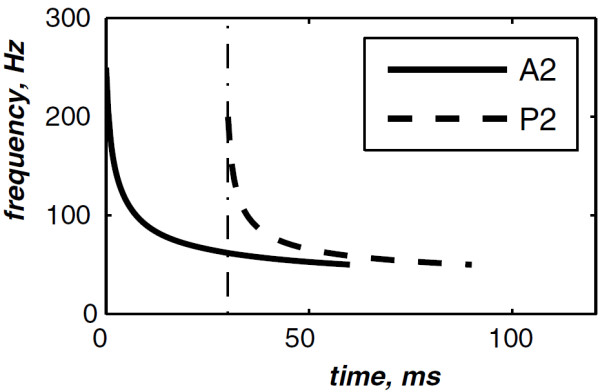
Instantaneous frequency of the simulated A2 (duration: 60 ms, frequency:250 →53 Hz) and P2 (duration: 60 ms, frequency: 200 →50 Hz) components of the S2 heart sound, A2–P2 valvular split: 30 ms.

**Figure 7:**
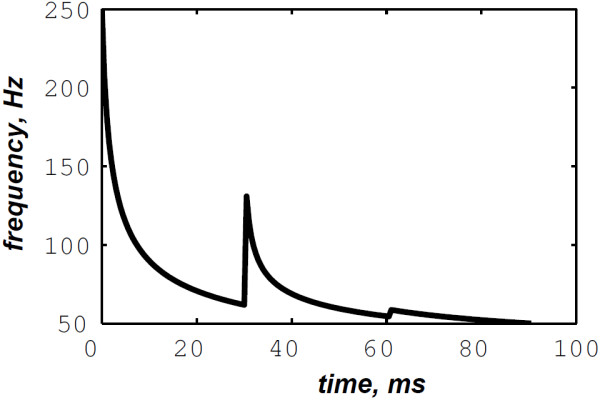
**Instantaneous frequency of the S2 heart sound (duration: 90 ms, A2–P2 valvular split: 30 ms), calculated from the IFs of the A2 and P2 valvular components of Figure **6**.**

**Figure 8:**
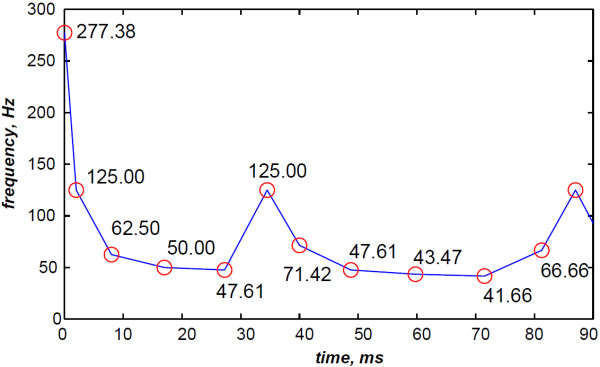
**Discrete IF of the envelope recovered S2 heart sound of Figure **9**.**

The simulated data are generated by the valvular heart sound model presented in section “Valvular heart sound model” [5,6]. According to (2), Figure 5 illustrates a simulated S2 heart sound with an A2–P2 split duration of 30 ms. The IF of the simulated S2 heart sound is illustrated in Figure 6. We processed the simulated S2 heart sounds at various valvular split durations (30, 40, 50 and 60 ms). Figure 7 shows the IF of the simulated S2 heart sound of Figure 5 with a A2–P2 split of 30 ms. This IF is the average of the IFs (Figure 6) of the A2 and P2 components which form the S2 heart sound (Figure 5). The discrete IF estimated by detecting the zero–crossings of S2 is illustrated in Figure 8. Both IFs show the inflection related to the valvular split around 30 ms at a high correlation level. The A2–P2 split is represented as a rapid transient during the merging interval between the aortic and the pulmonary chirp components within the time–frequency plane. As depicted in Figure 7, it should be noticed that the split occurs approximately between 50 and 150 Hz. The decreasing frequency of both the A2 and the P2 valvular components are adequately confirmed by the discrete IF plotted in Figure 8.

**Figure 9:**
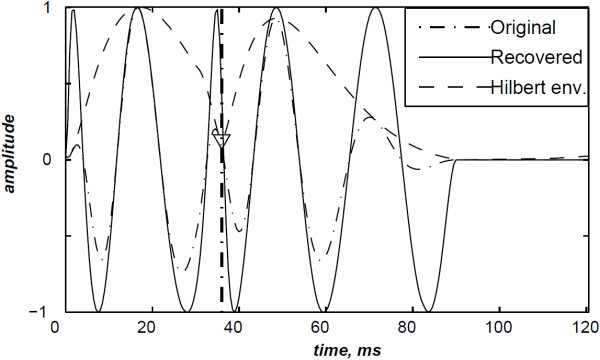
Envelope recovered S2 heart sound (detected A2–P2 valvular split: 36 ms).

To deal with the amplitude weakness of the valvular sounds encountered by Xu *et al.*[5] during time–frequency analysis of heart sounds, we calculated another version of heart sounds which we call the envelope recovered heart sound as presented in section “Detection algorithm of the A2–P2 valvular split”. Figure 9 illustrates the envelope recovered sound of the S2 heart sound of Figure 5. This adjusted sound benefits of the same amplitude for its entire time support.

The Hilbert envelope detection approach retraces the instantaneous power of this S2 heart sound and shows an amplitude variation which is related to the A2–P2 split. This split is localised at 36 ms from the onset of the A2 valvular component at an error of 6 ms from the original split (30 ms).

The recovery of the heart sound is carried out until reaching a cross–correlation between consecutive steps of 99.90% as presented in section “Detection algorithm of the A2–P2 valvular split”. This procedure of the A2–P2 split detection algorithm is not CPU time consuming and still a vital step for the time–frequency analysis. It should be noticed that 8 iterations are sufficient to reach the desired cross–correlation rate for this simulated S2 heart sound.

**Figure 10:**
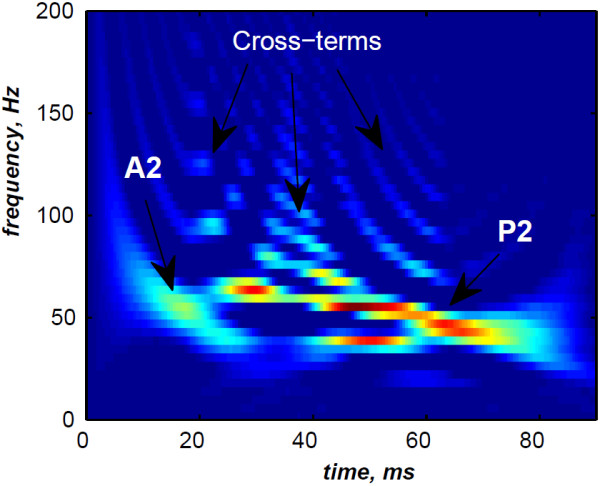
**WVD of the S2 heart sound of Figure **5**.**

As illustrated in Figure 10, the WVD of the S2 heart sound of Figure 5 is blurred by cross–terms. Moreover, the weakness of the A2 and P2 components at their onsets and their ends affects their respective time–frequency coefficients. Indeed, These components appears at their middle rather than the entire durations of each components.

**Figure 11:**
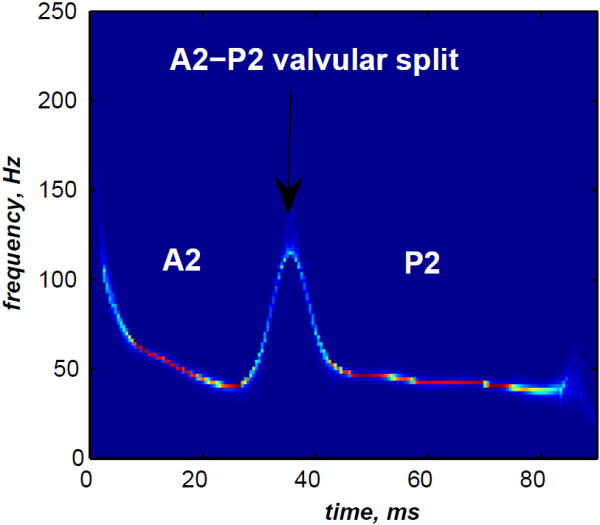
**RSPWVD of the S2 heart sound of Figure **9**.**

In contrast, the RSPWVD provides an extraordinary time–frequency representation which retraces perfectly the IF of the S2 heart sound. Indeed, as illustrated in Figure 11, the RSPWVD of the envelope recovered S2 heart sound of Figure 9 is highly correlated with the original IF of Figure 7 as well as the discrete IF of Figure 8. This reassigned distribution recovered the A2–P2 split around 30 ms and between 50 and 150 Hz as previously defined during the synthesis of the S2 heart sound (Figure 5).

**Figure 12:**
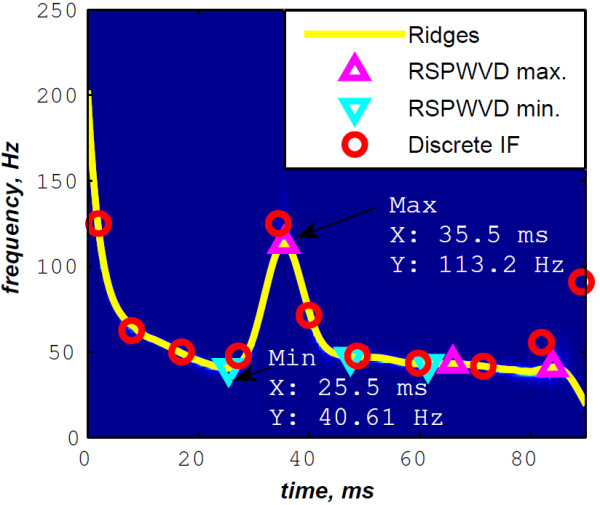
**Ridges (yellow line) of the RSPWVD of Figure **11** (detected A2–P2 valvular split:**$\frac{25.5+35.5}{2}=30.5$** ms).**

In Figure 12, the discrete IF of Figure 8 is represented with local extrema of the inflection zone within the RSPWVD. It should be noticed that the obtained RSPWVD retraces perfectly this discrete IF in the effective duration of the analysed S2 heart sound. Moreover, we detected the maximum intensity coefficients of the RSPWVD of Figure 12 to yield the IF of the analysed S2 heart sound. We also detected local maxima and minima of this curve to give an estimation of the A2–P2 valvular split by averaging time instants of the maximum and the minimum points at the inflection zone of the detected IF. According to this estimation approach, we found 30.5 ms as an A2–P2 valvular split in the RSPWVD of Figure 12.

The RSPWVD detection method is based on the variation of the IF of the S2 heart sound within the time–frequency plane rather than following variations in the amplitude of the signal as carried out by the Hilbert envelope detection approach. Therefore, the RSPWVD–based A2–P2 detection is accurate and improves the detection in comparison to the Hilbert envelope approach.

**Figure 13:**
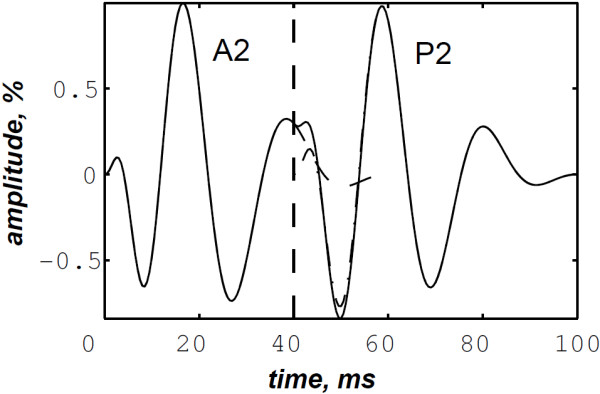
Simulated S2 heart sound, A2–P2 valvular split: 40 ms.

**Figure 14:**
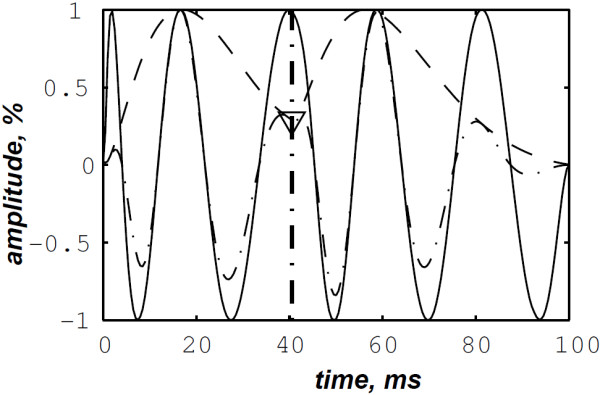
**Envelope recovered signal (solid line) of the S2 heart sound (dash–dot line) of Figure **13**and its Hilbert transform envelope (dashed line).** Valvular split: 40 ms.

**Figure 15:**
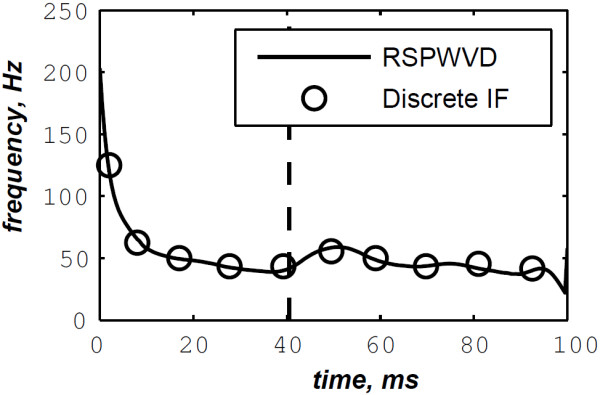
**RSPWVD of the envelope recovered S2 heart sound of Figure **14**(A2–P2 valvular split of 40 ms detected at 44 ms).**

**Figure 16:**
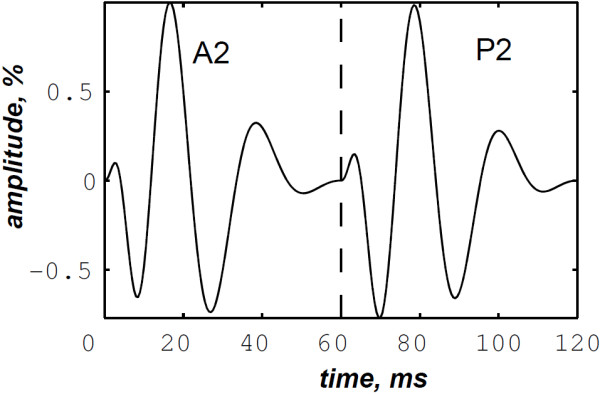
Simulated S2 heart sound, A2–P2 valvular split: 60 ms.

**Figure 17:**
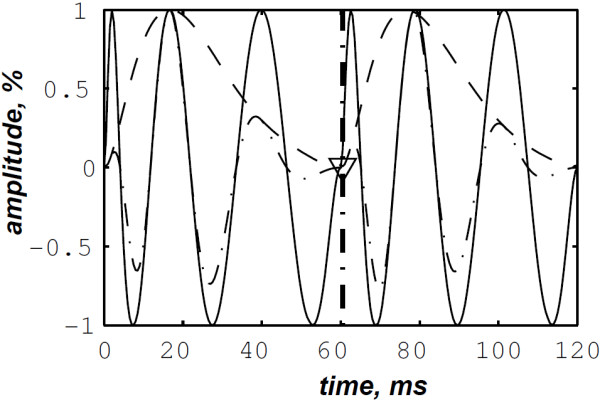
**Envelope recovered signal (solid line) of the S2 heart sound (dash–dot line) of Figure **16** and its Hilbert transform envelope (dashed line).**

**Figure 18:**
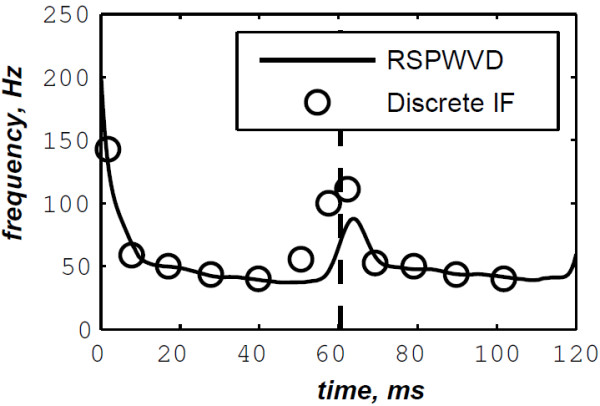
**RSPWVD of the envelope recovered S2 heart sound of Figure **17**(A2–P2 valvular split of 60 ms detected at 56. 25 ms).**

As depicted in Table 1, we extended the processing to various A2–P2 valvular split durations (30, 40, 50 and 60 ms). The simulated S2 heart sound splitted at 40 ms from its A2 component is depicted in Figure 13. Its envelope recovered version and its RSPWVD are illustrated in Figures 14 &15, respectively. Similarly, a simulated S2 sound splitted at 60 ms is illustrated in Figure 16, and its envelope recovered version as well as its RSPWVD are depicted in Figures 17 &18, respectively.

**Table 1:** The A2–P2 valvular split detected by the RSPWVD–based detection method

**Split (ms)**	**30**	**40**	**50**	**60**
A2–P2_min_	25.5	37	38.5	49
A2–P2_max_	35.5	51	52.5	63.5
A2–P2_mean_	30.5	44	45.5	56.25
A2–P2_error_	+0.5	+4	+4.5	-3.75

The A2–P2 split measurements summarised in Table 1 confirms the ability of the RSPWVD–based method to detect the A2–P2 split. The RSPWVDs of Figures 15 &18 continues to show the inflection behaviour at the A2–P2 split zone and provides 44 and 56.25 ms as measured values of the simulated split values of 40 and 60 ms.

### Detection of the A2–P2 valvular split in real S2 heart sounds of the LGB–IRCM cardiac valve database

The PCG signal of Figure 4 corresponding to the sample data file (10001.11) of the LGB–IRCM cardiac valve database is considered for analysis to show the ability of the proposed algorithm to detect the valvular split on real data. This PCG signal is segmented in systole and diastole by detecting the peak of the R–wave and the end of the T–wave by an algorithm presented in [39] which uses both the amplitude and the curvature of the ECG waves. This algorithm provides correct detection of the overall ECG–PCG signals of the LGB–IRCM cardiac valve database.

**Figure 19:**
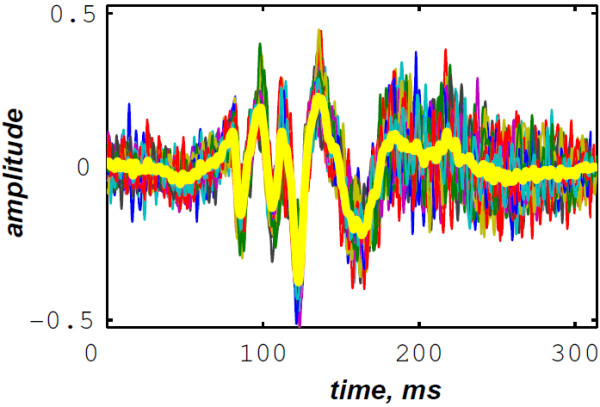
LGB–IRCM cardiac valve database (data file: 10001.11): averaged S1 (yellow line) over 40 systolic phases (coloured lines).

**Figure 20:**
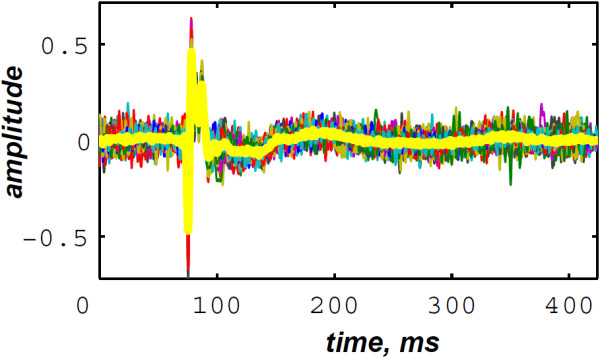
LGB–IRCM cardiac valve database (data file: 10001.11): averaged S2 (yellow line) over 40 diastolic phases (coloured lines).

Figures 19 &20 illustrate respectively the averaged S1 and S2 heart sounds of the PCG signal of the LGB–IRCM data file 10001.11 (Figure 4). The main advantage behind averaging the PCG segments over the consecutive cardiac cycles is to fade the background noise to yield a smooth signal for the subsequent processing. The S2 heart sounds are adjusted according to their recurrent autocorrelation functions over the consecutive cardiac cycles. This adjustment resolves the jitter of the S2 heart sound within the diastole phase and keeps up the aortic and the pulmonary chirp components for the subsequent time–frequency analysis.

**Figure 21:**
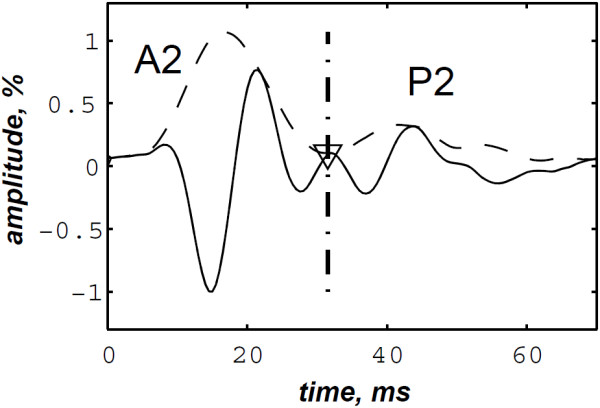
LGB–IRCM cardiac valve database (data file: 10001.11): averaged S2 over 40 cardiac cycles and its Hilbert transform envelope (dashed line).

**Figure 22:**
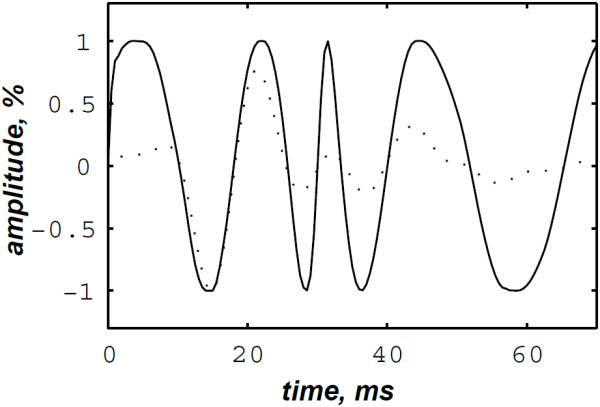
**LGB–IRCM cardiac valve database (data file: 10001.11): envelope recovered S2 heart sound (solid line) of the S2 heart sound (dotted line) of Figure **21**.**

As depicted in Figure 21, the S2 heart sound averaged in Figure 20 is formed by two chirps; namely the A2 and the P2 valvular components. The A2 chirp is of higher amplitude than that of the P2 component which confirms the recording carried out from the aortic auscultation area. The envelope recovered version of this S2 heart sound illustrated in Figure 22 obviously highlights the frequency modulated behaviour of the valvular sound.

**Figure 23:**
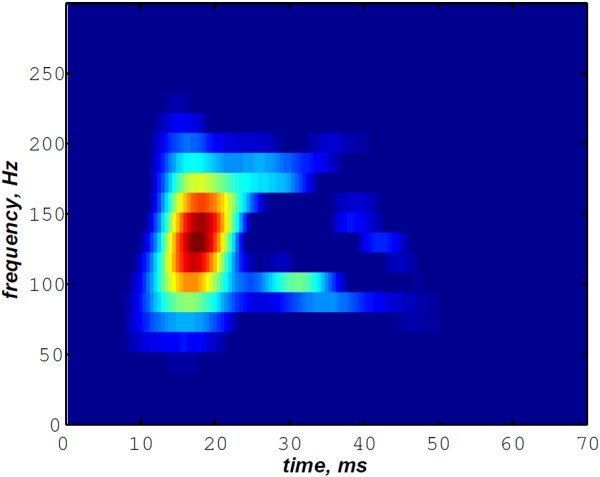
**WVD of the S2 heart sound of Figure **22**.**

The WVD of Figure 23 of the averaged S2 heart sound of Figure 21 is concentrated within the time support of the A2 valvular chirp which is of highest amplitude than that of the P2 component. The split behaviour we found in section “Detection of the A2–P2 valvular split in simulated heart sounds” is not reproduced by the WVD. As demonstrated by Xu *et al.*, the weakness of the valvular sounds at their extremities restricts the energy bursts in the high amplitude time support of the analysed sounds within the time–frequency plane [5].

**Figure 24:**
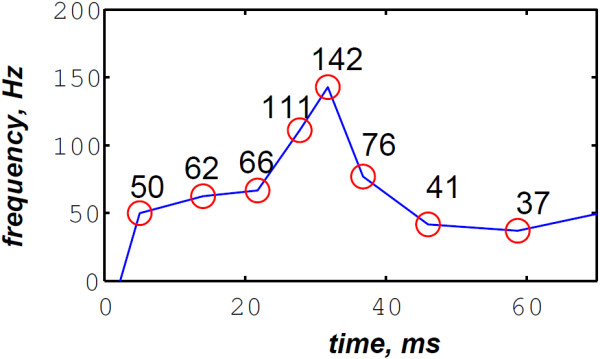
**Discrete IF of the envelope recovered S2 heart sound of Figure **22**.**

**Figure 25:**
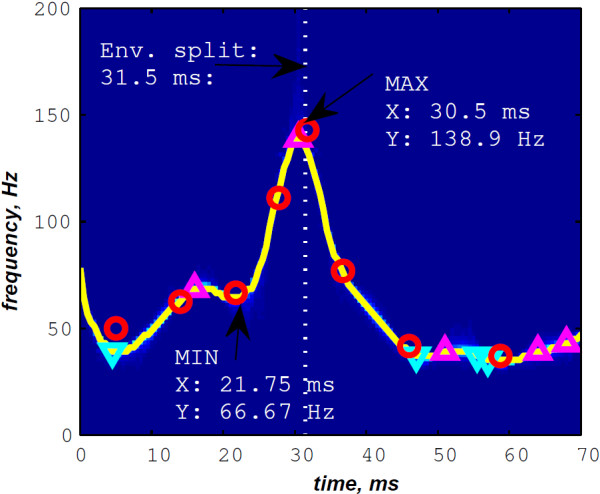
**RSPWVD of the envelope recovered S2 heart sound of Figure **22**and the discrete IF of Figure **24** (circles plot).**

The discrete IF of Figure 24, which is calculated from the envelope recovered S2 heart sound of Figure 22, is adequately retraced by the RSPWVD of Figure 25. This RSPWVD resolved the A2–P2 valvular split of the real S2 heart sound at a high time and frequency resolutions. Indeed, the time–frequency representation illustrated in Figure 25 yields a similar result in comparison to the simulations carried out in section “Detection of the A2–P2 valvular split in simulated heart sounds”. The A2 and P2 valvular chirps are clearly reproduced at a high correlation with the simulated heart sounds as well as their split within the S2 heart sound. similarly, the valvular split in the real S2 heart sound is also localised between 50 and 150 Hz. As depicted in Figure 25, if we take the average between the time instants of the maximum (30.5 ms) and the minimum (21.75 ms) frequencies during as an estimation of the A2–P2 valvular split, we obtain 26.125 ms which is confirmed by the discrete IF illustrated in Figure 24.

## Conclusions

The A2–P2 valvular split detection algorithm we developed is mainly based on the Reassigned Smoothed Pseudo Wigner–Ville Distribution (RSPWVD). The Reassignment mixed to the smoothing achieved both in time and frequency domains by the SPWVD provides a higher readability to the obtained RSPWVD. The preprocessing envelope recovery procedure we proposed adapts the analysed heart sounds to the WVD which is optimal for analysing frequency modulated chirps. The performance of the algorithm is confirmed on simulated heart sounds at various split durations (30, 40, 50 and 60 ms). The A2–P2 valvular split is localised at the frequency inflection in the obtained RSPWVD. The developed algorithm is validated on real heart sounds of the LGB–IRCM cardiac valve database and retraces the inflection of the A2–P2 valvular split of the S2 heart sound within the time–frequency plane. The discrete IF is estimated for both simulated and real data to confirm the results obtained through the RSPWVD. Therefore, the proposed algorithm deals adequately with detecting the A2–P2 valvular split and confirms the chirp behaviour of heart sounds. Thus, we demonstrated through the algorithm we developed that the A2–P2 valvular split can be accurately detected by time–frequency analysis using the RSPWVD.

## Consent

The patients were contacted in confidentiality by their treating cardiologist and they signed an inform consent form attesting their assent to take part in the study allowing to record the LGB–IRCM cardiac valve database. The signals were recorded at the Institut de recherches cliniques de Montréal (IRCM) and at the Montreal General Jewish Hospital in Quebec (Canada).

## Appendix: Properties of the Wigner–Ville distribution

The WVD has some nice properties which are summarised as follows. 

1. The WVD is a member of the Cohen’s class with a weighting function *g*(*ν*,*τ*) = 1.

2. Realness: The WVD is of real values over the time–frequency plane which makes it suitable for representing the energy of the analysed signal.

3. Time and frequency marginals: As shown in (13), the integration of the time–frequency distribution over time yields the spectral density of the signal. As stated in (14), the integration of the time–frequency distribution over frequency yields the instantaneous power of the analysed signal *x*(*t*), as follows;

(13) $$\overset{+\infty }{\underset{-\infty }{\int }}W_{x}(t,f)\;dt=|X(f){|}^{2}$$

(14) $$\overset{+\infty }{\underset{-\infty }{\int }}W_{x}(t,f)\;df=|x(t){|}^{2}$$

where *X*(*f*) denotes the Fourier transform of the signal *x*(*t*), and *W*_*x*_(*t*,*f*) represents its WVD.

4. Global energy: Integration of the WVD over the time–frequency plane yields the global energy *E*_*x*_ of the analysed signal as follows;

(15) $$\overset{+\infty }{\underset{-\infty }{\iint }}W_{x}(t,f)\;dt\;df=E_{x}$$

5. Instantaneous frequency (IF): the first moment of the WVD with respect to frequency of the analytic signal yields the IF as follows;

(16) $$\frac{\overset{+\infty }{\underset{-\infty }{\int }}fW_{x}(t,f)\;df}{\overset{+\infty }{\underset{-\infty }{\int }}W_{x}(t,f)\;df}=\frac{1}{2\pi }\;\frac{d}{dt}[\text{arg}x(t)]$$

6. Time delay (TD): the first moment of the WVD with respect to time of the analytic signal of yields the TD as follows;

(17) $$\frac{\overset{+\infty }{\underset{-\infty }{\int }}tW_{x}(t,f)\;dt}{\overset{+\infty }{\underset{-\infty }{\int }}W_{x}(t,f)\;dt}=-\frac{1}{2\pi }\;\frac{d}{df}[\text{arg}X(f)]$$

7. The WVD is limited to time–frequency support defined by the duration and the bandwidth of the analysed signal *x*(*t*).

8. Convolution invariance: The WVD of the time–convolution of two signals (*x*_1_(*t*)and*x*_2_(*t*)), yields the time– convolution of their respective WVDs$\left(W_{x_{1}}(t,f)\text{and}W_{x_{2}}(t,f)\right)$ as follows;

(18) $$\begin{array}{lll} x_{3}(t) & =x_{1}(t)\underset{t}{*}x_{2}(t)\; & \\ \Rightarrow W_{x_{3}}(t,f) & =W_{x_{1}}(t,f)\underset{t}{*}W_{x_{2}}(t,f)\; \end{array}$$

9. Modulation invariance: The WVD of the frequency–convolution of two signals (*x*_1_(*t*) and*x*_2_(*t*)) yields the frequency–convolution of their respective WVDs$\left(W_{x_{1}}(t,f)\text{and}W_{x_{2}}(t,f)\right)$ as follows;

(19) $$\begin{array}{lll} x_{3}(t) & =x_{1}(t)\cdot x_{2}(t)\; & \\ \Rightarrow W_{x_{3}}(t,f) & =W_{x_{1}}(t,f)\underset{f}{*}W_{x_{2}}(t,f)\; \end{array}$$

The WVD is time and frequency invariant. Furthermore, a range of peaks of the IF and TD of the analysed signal are directly readable on the WVD [40,41]. The WVD covers the spectral bandwidth of the analysed signal. Moreover, fluctuations of the maximum frequency of the analysed signal is well represented over the time domain by the WVD [42].

## Competing interests

The authors declare that they have no competing interests.

## Authors’ contributions

AD has simulated the second heart sound by a chirp model, estimated its instantaneous frequency, calculated the corresponding zero–crossing instantaneous frequency, calculated the valvular split within the second heart sound, proposed the new envelope recovery method to improve the time–frequency analysis of heart sounds, developed a new algorithm based on the Reassigned Smoothed Pseudo Wigner–Ville Distribution (RSPWVD) to detect the valvular split within heart sounds, applied the time–frequency analysis on simulated and real heart sounds, developed the MATLAB code, and written the manuscript. FBR has revised the manuscript, and reviewed the results. Both authors read and approved the final manuscript.
